# The bisphosphonate pamidronate induces apoptosis in human melanoma cells *in vitro*

**DOI:** 10.1038/sj.bjc.6600476

**Published:** 2002-08-01

**Authors:** C Riebeling, A-M Forsea, M Raisova, C E Orfanos, C C Geilen

**Affiliations:** Department of Dermatology, University Medical Center Benjamin Franklin, The Free University of Berlin, Fabeckstr. 60-62, 14 195-Berlin, Germany

**Keywords:** bisphosphonates, pamidronate, Rho proteins, melanoma, apoptosis

## Abstract

Pamidronate belongs to the class of nitrogen-containing bisphosphonates that are potent inhibitors of bone resorption frequently used for the treatment of osteoporosis and cancer-induced osteolysis. The inhibition of osteoclasts’ growth has been suggested as the main mechanism of the inhibitory effect of pamidronate on bone metastases. Recent findings indicated that bisphosphonates also have a direct apoptotic effect on other types of tumour cells. Nitrogen-containing bisphosphonates were shown to inhibit farnesyl diphosphate synthase, thus blocking the synthesis of higher isoprenoids. By this mechanism they inactivate monomeric G-proteins of the Ras and Rho families for which prenylation is a functional requirement. On the background of the known key role of G-proteins in tumorigenesis, we investigated a possible beneficial use of pamidronate in the treatment of malignant melanoma. Our results indicate that pamidronate inhibits the cell growth and induces apoptosis in human melanoma cells *in vitro*. Susceptibility to pamidronate did not correlate to CD95 ligand sensitivity or p53 mutational status. Furthermore it is interesting to note that overexpression of bcl-2 did not abolish pamidronate-induced apoptosis. These data suggests that pamidronate has a direct anti-tumour effect on malignant melanoma cells, independently of the Bax/Bcl-2 level.

*British Journal of Cancer* (2002) **87**, 366–371. doi:10.1038/sj.bjc.6600476 www.bjcancer.com

© 2002 Cancer Research UK

## 

At present, melanoma belongs to the most malignant tumours of the skin and mucous membranes due to its aggressive biological behaviour and tendency to generate early metastases. Malignant melanoma is characterised by its relatively high therapeutical resistance (Serrone and Hersey, 1999). Therefore, new therapeutical strategies need to be established.

Monomeric G-proteins of the Ras and Rho families have been shown to be involved in tumorigenesis and metastasis. Both Ras and Rho GTPases are involved in key cellular processes, such as reorganisation of the cytoskeleton, membrane trafficking, lipid metabolism, transcriptional regulation, cell growth, and apoptosis (Aznar and Lacal, 2001). However, overexpression or constitutive activation caused by mutations, rendering them insensitive to regulatory signals reveal the negative aspects of these multifunctional proteins. The dysregulation of these GTPases triggers specific signals that provoke uncontrolled cell growth, enhanced angiogenesis, inhibition of apoptosis, and genetic instability, all main aspects of tumour development. Moreover, Rho family proteins are engaged in regulation of the cytoskeleton and remodelling of cytoarchitecture, which is a requirement of metastasis. Interestingly, enhanced expression of several Rho family members was observed in metastatic tumour cells (Suwa *et al*, 1998; Fritz *et al*, 1999; Clark *et al*, 2000). Recently, it was described that ectopic overexpression of RhoC in A375 melanoma cell line was sufficient to create a highly metastatic phenotype (Clark *et al*, 2000).

For monomeric G-proteins of the Ras and the Rho families prenylation is a functional requirement. The members of the Ras superfamily are acylated and/or prenylated; the acyl/prenyl residues function as membrane anchors when the G-proteins are released from their complex with a guanine nucleotide dissociation inhibitor. Recently, nitrogen-containing bisphosphonates were shown to specifically inhibit farnesyl diphosphate synthase (van Beek *et al*, 1999; Bergstrom *et al*, 2000), the enzyme that executes the initial condensation of isopentenyl diphosphate and dimethylallyl diphosphate thereby inhibiting protein prenylation (Luckman *et al*, 1998). Thus, nitrogen-containing bisphosphonates interfere with membrane localization of these proteins and may abolish signal-mediation which can be of use in countering effects of their overexpression or mutation.

Bisphosphonates are used for the treatment of bone metastases and were initially thought to act via an inhibition of formation of osteoclasts from immature precursor cells or direct inhibition of resorption via induction of apoptosis in mature osteoclasts (Hughes *et al*, 1995). Recently, evidence accumulated that bisphosphonates including pamidronate are potent inducers of apoptosis in several cancer cell types such as myeloma (Shipman *et al*, 1997; Aparicio *et al*, 1998), breast cancer (Senaratne *et al*, 2000) and prostate cancer (Lee *et al*, 2001) but also in macrophage cell lines (Rogers *et al*, 1996) and epithelial cell lines (Twiss *et al*, 1999). These data indicate that the beneficial effect of bisphosphonates may result from a direct anti-tumour activity that may affect a broad range of metastasing tumours.

In the present study, we demonstrate that micromolar concentrations of pamidronate inhibit cell proliferation and induce apoptosis in different melanoma cell lines. The susceptibility to CD95 ligand or the mutational status of p53 did not correlate with the sensitivity to pamidronate. Moreover, overexpression of bcl-2 did not rescue the cells from pamidronate-induced apoptosis. Nevertheless, the apoptotic effect can be partially abolished by coincubation with farnesol or geranylgeraniol confirming the specific effect of pamidronate on inhibition of prenylation.

## MATERIALS AND METHODS

### Materials

Pamidronate (3-amino-1-hydroxy-propylidene-1,1-bisphosphonate) was purchased from Novartis Pharmaceuticals Limited (Basel, Switzerland). Anti-caspase-3 antibodies (rabbit) were obtained from Pharmingen (Hamburg, Germany) and horseradish peroxidase-conjugated secondary antibodies were purchased from Dako (Hamburg, Germany). Farnesol and geranylgeraniol were obtained from Sigma (Munich, Germany).

### Cell culture

A375 cells (Giard *et al*, 1973) originate from primary tumour and Mel2A (Bruggen *et al*, 1981), MeWo (Bean *et al*, 1975) and SkMel23 (Carey *et al*, 1976) originate from metastases. The two melanoma cell populations M186 and M221 were obtained from patients with histologically confirmed metastatic melanoma by surgical intervention. The generation and functionality of A375/pIRES and A375/mbcl-2 was described recently (Raisova *et al*, 2001). Melanoma cells were grown in 90% Dulbecco's Modified Eagle's Medium (DMEM), 10% heat-inactivated foetal calf serum and supplemented with penicillin (100 IU ml^−1^) and streptomycin (100 μg ml^−1^) in 75 cm^2^ tissue culture flasks (Nunc, Wiesbaden, Germany). Prior to the experiments, the cell lines were cultured for five passages under the same seeding and growth conditions so as to achieve a comparable growth behaviour. DMEM was purchased from Life Technologies (Eggenstein, Germany); further cell culture reagents were obtained from Seromed-Biochrom (Berlin, Germany).

### Cytotoxicity assay

Cytotoxicity of pamidronate was determined by the release of lactate dehydrogenase from the cytosol of damaged cells using an LDH cytotoxicity detection kit (Roche Diagnostics, Mannheim, Germany). Melanoma cells were seeded at a density of 40 000 cells per cm^2^ and after 36 h treated with agents as indicated or corresponding solvents as control. After incubation, the cell culture supernatant was removed and clarified by centrifugation at 400 **g** for 5 min. Of the supernatants, 50 μl were transferred to microtiter plates and diluted with 50 μl of PBS. After addition of 100 μl reaction mixture containing 2-[4-iodophenyl]-3-[4-nitrophenyl]-5-phenyl tetrazolium chloride, diaphorase, NAD^+^ and sodium lactate, the samples were incubated for 30 min at room temperature, protected from light. The extinction at 492 nm was measured using an ELISA reader. The extinction values of control cells were set as 100% and the rate of lactate dehydrogenase release from treated cells was calculated as per cent of control.

### Cell proliferation assay

Melanoma cells were seeded at a density of 20 000 cells per cm^2^ and after 36 h treated with agents as indicated or corresponding solvents as control. Cell proliferation was determined by staining cells with crystal violet as described (Wieder *et al*, 1998). Briefly, non-adherent cells were washed off and the remaining cells were fixed with 0.1 M glutaraldehyde in PBS for 30 min. After washing, the cells were incubated in 0.2 mM crystal violet in PBS for 30 min. Unbound dye is washed off and 0.2% Triton X-100 is added to release bound dye. The extinction of the supernatants at 570 nm was measured using an ELISA reader. The extinction values of vehicle-treated control cells were set as 100% and the rate of proliferation of treated cells was calculated as per cent of control.

### Cell death detection ELISA

Melanoma cells were seeded at a density of 40 000 cells per cm^2^ and after 36 h treated with agents as indicated or corresponding solvents as control. Apoptosis was determined quantitatively by using the Cell Death Detection ELISA^Plus^ Assay (Roche Diagnostics, Mannheim, Germany). Cells were centrifuged at 150 **g** for 5 min and the supernatant was aspirated. Lysis buffer was added and the cells incubated for 30 min. Then, a clear cytosolic supernatant was prepared by centrifugation at 150 **g** for 10 min. Of the respective supernatants, 20 μl were added to a streptavidin-coated microtiter plate. After addition of 80 μl of a solution containing anti-histon-biotin antibodies and anti-DNA-peroxidase antibodies in incubation buffer, the samples were incubated under moderate shaking for 2 h. Subsequently, the wells were washed three times with incubation buffer, 100 μl of substrate solution were added and the plate was incubated for up to 10 min at room temperature in the dark. The absorption was measured at 405 nm using an ELISA reader. The values of control cells were set as 100% and the apoptosis rates of treated cells were calculated as per cent of control.

### Immunoblotting

Cells were seeded at a density of 40 000 cells per cm^2^ in 25 cm^2^ flasks and after 36 h treated with agonist or corresponding solvents for controls as stated. Subsequently, cells were scraped on ice in 150 μl of 50 mM Tris·HCl, pH 7.2, 150 mM NaCl, 1 mM EDTA, 1% Triton X-100, 3.5 mM SDS, 1 mM PMSF, 10 μM leupeptin and 1 μM pepstatin. After homogenisation by sonication for 10 s, samples were centrifuged for 10 min with 400 **g** at 4°C. Aliquots of the supernatant were mixed with SDS sample buffer (Laemmli, 1970) containing 0.1 M DTT instead of β-mercaptoethanol and denatured for 5 min at 95°C. After separation by sodium dodecyl sulphate-polyacrylamide gel electrophoresis (SDS–PAGE) and transfer to nitrocellulose membrane the blot was incubated in PBS containing 3% non-fat dried milk and 0.1% Tween 20 for 20 min to block nonspecific binding. The membrane was then incubated with anti-caspase-3 antibodies 1 : 2000 in blocking buffer for 2 h. Bound antibodies were detected after washing using 50 ng ml^−1^ horseradish peroxidase-conjugated secondary antibodies in blocking buffer and the SuperSignal chemiluminescent detection reagent (Pierce, Weiskirchen, Germany). Protein was measured in samples diluted 1 : 5 in H_2_O using the BCA assay (Pierce, Weiskirchen, Germany) with bovine serum albumin as standard.

## RESULTS

### Inhibition of melanoma cell proliferation by pamidronate

**Figure 1:**
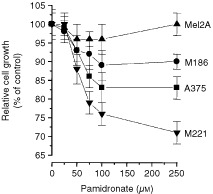
Effect of pamidronate on melanoma cell proliferation. A375, M186, Mel2A and M221 melanoma cells were seeded at a density of 20 000 cells per cm^2^ and after 36 h treated with indicated concentrations of pamidronate or vehicle control for 48 h. Proliferation was measured using crystal violet staining of adherent cells. Cell growth of control cells was set as 100% and proliferation of treated cells was calculated as a percentage of control. Values represent the mean of four experiments±s.d.

To evaluate the effect of pamidronate on melanoma cells, four different cell populations were chosen. A375 and M186 are confirmed to be sensitive to CD95 ligand as well as to ceramide whereas Mel2A and M221 are resistant to these stimuli (Raisova *et al*, 2000). Treatment with increasing concentrations of pamidronate resulted in a concentration-dependent decrease in cell number as assessed after 48 h (Figure 1

). The greatest reduction in cell numbers was observed at a concentration of 100 μM pamidronate for A375, M186 and M221. Cell numbers were significantly reduced in the CD95 ligand-sensitive cell lines A375 with 83±4% of control and M186 with 89±4% of control as well as in the CD95 ligand-resistant M221 with 76±4% of control. Cytotoxicity was excluded by measurement of LDH release into the cell culture supernatant. Higher concentrations of 250 μM pamidronate did not further reduce cell numbers. In contrast, the proliferation of the CD95 ligand-resistant cell line Mel2A was not affected by pamidronate at any of the used concentrations. These data suggest that the observed effect on cell number is due to growth inhibition and not primary cytotoxicity of pamidronate.

### Apoptosis in melanoma cells after pamidronate treatment

**Figure 2:**
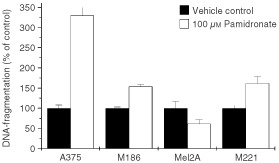
Apoptosis of melanoma cells after pamidronate treatment. A375, M186, Mel2A and M221 melanoma cells were seeded at a density of 40 000 cells per cm^2^ and after 36 h treated with vehicle control or 100 μM pamidronate for 24 h. DNA-fragmentation was quantified using an enzyme-linked immunoassay detecting cytoplasmic nucleosomes. DNA-fragmentation of control cells was set as 100% and DNA-fragmentation of treated cells was calculated as a percentage of control. Values represent the mean of four experiments±s.d.

One of the key features of apoptosis is the controlled degradation of nuclear DNA, resulting in histone-bound DNA fragments released into the cytoplasm of the cell. An ELISA assay was used to measure these nucleosomes to achieve a quantification of apoptosis. Treatment of melanoma cells with different concentrations of pamidronate for 24 h led to a concentration-dependent increase of DNA-fragmentation. As observed for inhibition of proliferation, the effect was greatest with 100 μM pamidronate. The CD95 ligand-sensitive cell lines A375 and M186 showed an increase of DNA-fragmentation after treatment with 100 μM pamidronate for 24 h to 330±20% of control and 154±5% of control, respectively (Figure 2

).

Induction of DNA-fragmentation was also shown in the CD95 ligand-resistant cell line M221 with 162±16% of control. However, in agreement with the absence of an effect on cell number, the CD95 ligand-resistant cell line Mel2A was also unaffected by pamidronate treatment in respect to DNA-fragmentation.

**Figure 3:**
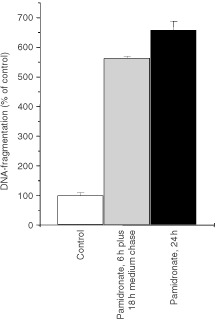
Apoptosis after short term exposure of A375 cells to pamidronate. A375 melanoma cells were seeded at a density of 40 000 cells per cm^2^ and the next day treated with vehicle or 100 μM pamidronate. Cells were either treated for 6 h with pamidronate, after which drug-containing medium was replaced with fresh culture medium, and the cells were further incubated for another 18 h or incubated with the drug containing medium for 24 h. Apoptosis was then measured as DNA-fragmentation using an enzyme-linked immunoassay detecting cytoplasmic nucleosomes. DNA-fragmentation of control cells was set as 100% and DNA-fragmentation of treated cells was calculated as a percentage of control. Values represent the mean of four experiments±s.d.

In contrast to pamidronate, clodronate, a non-amino bisphosphonate showed no effect on proliferation and apoptosis induction in melanoma cell lines at the same concentration range (data not shown). It is interesting to note that incubation of melanoma cells with 100 μM pamidronate for only 6 h, followed by an 18 h medium chase, resulted in nearly the same apoptotic effect as the 24 h incubation with the drug (Figure 3

). This short time effect would correspond to the peak levels of circulating bisphosphonate observed in clinical practice.

**Figure 4:**
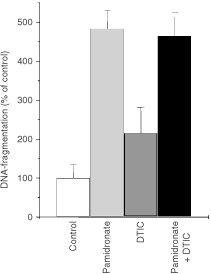
Effect of combination of pamidronate with DTIC. A375 melanoma cells were seeded at a density of 40 000 cells per cm^2^ and the next day treated with 100 μM Pamidronate, 5 μg ml^−1^ DTIC or both. After 24 h of incubation apoptosis was measured. DNA-fragmentation of control cells was set as 100% and DNA-fragmentation of treated cells was calculated as a percentage of control. Values represent the mean of four experiments±s.d.

In order to investigate a possible synergistic effect of pamidronate and DTIC, a standard chemotherapeutical agent used in melanoma, A375 and M186 melanoma cell lines were incubated for 24 h with 100 μM pamidronate, with 5 μg ml^−1^ DTIC and with pamidronate plus DTIC, respectively. These experiments indicate that 100 μM pamidronate has a greater proapoptotic effect than 5 μg ml^−1^ DTIC. However, the combination of both compounds failed to show any synergistic or additive effect in apoptosis induction. The results were similar in the two cell lines studied; the results in A375 are shown in Figure 4

.

### Caspase-3 is activated in pamidronate-induced apoptosis

**Figure 5:**
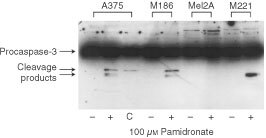
Procaspase-3 cleavage upon pamidronate treatment in melanoma cells. A375, M186, Mel2A and M221 melanoma cells were seeded at a density of 40 000 cells per cm^2^ and after 36 h treated with 100 μM pamidronate (+), vehicle control (−) or 1 μg ml^−1^ of the CD95-agonistic monoclonal antibody CH-11 (C) as a positive control for 24 h. Whole cell lysates were prepared and 50 μg of each were separated on a 15% SDS–PAGE. After transfer to nitrocellulose the membrane was probed with anti caspase-3 antibodies recognising procaspase-3 and cleavage products forming active caspase-3 respectively. Immunocomplexes were detected using Super Signal chemiluminescence reagent.

Activation of the executor-caspases -3, -6 or -7 is a further key-step in apoptosis. Their activation by proteolytic processing is the point-of-no-return in the commitment to apoptosis. Western blot was performed using anti caspase-3 antibodies that detect the proenzyme and cleaved enzyme forms. Cells were treated with 100 μM pamidronate or vehicle for 24 h and in case of A375 with the CD95-agonistic monoclonal antibody CH-11 as a positive control. Activation of procaspase-3 is detected by appearance of cleaved forms of 20 and 17 kDa. The CD95 ligand-sensitive cell lines A375 and M186 show cleaved forms after pamidronate-treatment but not in vehicle controls. This is confirmed by cleavage of procaspase-3 after treatment of A375 with CH-11. M221, a CD95 ligand-resistant cell line exhibits cleaved forms of caspase-3 after pamidronate-treatment. Furthermore, in correlation to the results for cell proliferation and DNA-fragmentation, the CD95 ligand-resistant cell line Mel2A shows no activation of caspase-3 (Figure 5

).

### p53 mutation or bcl-2 overexpression do not abolish pamidronate-induced apoptosis

**Figure 6:**
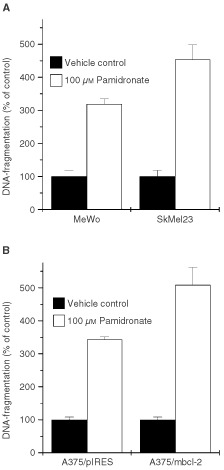
Induction of apoptosis in p53-mutated and bcl-2 overexpressing melanoma cells by pamidronate. (**A**) The p53-mutated cell lines MeWo and SkMel23 and (**B**) the mock-transfected A375/pIRES and the bcl-2 overexpressing A375/mbcl-2 were seeded at a density of 40 000 cells per cm^2^ and after 36 h treated with vehicle control or 100 μM pamidronate for 24 h. DNA-fragmentation was quantified using an enzyme-linked immunoassay detecting cytoplasmic nucleosomes. DNA-fragmentation of control cells was set as 100% and DNA-fragmentation of treated cells was calculated as a percentage of control. Values represent the mean of four experiments±s.d.

Two frequent mechanisms by which tumour cells achieve chemoresistance were tested for their effect on pamidronate-induced apoptosis. The two melanoma cell lines MeWo and SkMel23 harbour a mutated p53 gene (Raisova *et al*, 2001). Mutations in this gene can render tumour cells insensitive to radiotherapy and chemotherapy. In agreement, these cell lines were shown to be resistant to CD95- and ceramide-induced apoptosis (Raisova *et al*, 2001). The cells were treated for 24 h with 100 μM pamidronate and apoptosis was quantified in terms of DNA-fragmentation. Both cell lines were sensitive to pamidronate treatment with DNA-fragmentation of 320±20% of control and 450±50% of control for MeWo and SkMel23, respectively (Figure 6A

).

Shifting the ratio of the proapoptotic protein bax to the antiapoptotic protein bcl-2 is another mechanism by which tumour cells escape the immune system and achieve resistance against drugs entering the mitochondrial apoptotic pathway. This resistance can be mimicked in cell culture by ectopic overexpression of bcl-2. A375 cells stably overexpressing murine bcl-2α (A375/mbcl-2) and A375 transfected with vector only (A375/pIRES) as control were treated for 24 h with 100 μM pamidronate. A375/mbcl-2 in contrast to A375/pIRES were shown to be resistant to CD95- as well as ceramide-induced apoptosis (Raisova *et al*, 2001). However, 100 μM pamidronate induced DNA-fragmentation in A375/pIRES with 343±8% of control as well as in A375/mbcl-2 with 509±53% of control (Figure 6B).

### Geranylgeraniol abolishes pamidronate-induced apoptosis

**Figure 7:**
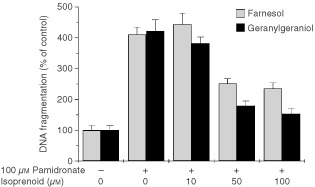
Effect of isoprenoids on pamidronate-induced apoptosis in melanoma cells. A375 melanoma cells were seeded at a density of 40 000 cells per cm^2^ and after 36 h treated with 100 μM pamidronate in the presence of indicated concentrations of farnesol, geranylgeraniol or vehicle control for 24 h. DNA-fragmentation was quantified as cytoplasmic nucleosomes using an enzyme-linked immunoassay. DNA-fragmentation of control cells was set as 100% and DNA-fragmentation of treated cells was calculated as a percentage of control. Values represent the mean of four experiments±s.d.

The most prominent effect of pamidronate is the inhibition of farnesyl diphosphate synthase, abolishing all isoprenoid biosynthesis (van Beek *et al*, 1999). To specifically circumvent the effect of pamidronate on isoprenoid biosynthesis, cells were supplemented with farnesol or geranylgeraniol. Farnesol can be used as a precursor for synthesis of all higher isoprenoids and protein-farnesylation whereas geranylgeraniol only supplies ubiquinone biosynthesis and protein-geranylgeranylation. The addition of increasing concentrations of either farnesol or geranylgeraniol in the presence of pamidronate reduced pamidronate-induced DNA-fragmentation in A375 cells in a concentration-dependent manner (Figure 7

). Farnesol or geranylgeraniol at a concentration of 10 μM had no impact on pamidronate-induced apoptosis. At a concentration of 50 μM, farnesol reduced the apoptotic effect of pamidronate by about 50% and 50 μM geranylgeraniol reduced DNA-fragmentation induced by pamidronate by 75%. However, higher concentrations of 100 μM only slightly further decreased pamidronate-induced apoptosis.

## DISCUSSION

In this study, we demonstrate that treatment of melanoma cell lines with the nitrogen-containing bisphosphonate pamidronate *in vitro* induces apoptosis and inhibits proliferation of human melanoma cells in a concentration-dependent manner. In contrast, clodronate, a non-amino bisphosphonate, had no effect in melanoma cell lines at the same concentration range. Furthermore, induction of apoptotic DNA-fragmentation was paralleled by caspase-3 cleavage in these cell lines. The CD95 ligand-sensitive cell lines A375 and M186 as well as the CD95 ligand-resistant cell lines M221, MeWo and SkMel23 showed DNA-fragmentation upon treatment with 100 μM pamidronate. This concentration of pamidronate has also been shown to induce apoptosis in other cell lines, e.g. myeloma (Shipman *et al*, 1997), breast cancer (Senaratne *et al*, 2000), and prostate cancer (Lee *et al*, 2001). However, the CD95 ligand-resistant cell line Mel2A was not affected by this bisphosphonate. A specific apoptotic effect of pamidronate was further confirmed by the observation that caspase-3 is cleaved and therefore activated in pamidronate-treated cells. Again, Mel2A showed no caspase-3 processing upon treatment with pamidronate. MeWo and SkMel23 harbour a mutated p53 gene that is a rather rare event in melanoma. Other tumours show frequent mutation in this gene, which often leads to resistance against chemotherapy. The susceptibility of these cell lines to pamidronate suggests a p53-independent pathway of apoptosis-induction for this drug. Experimental settings using bcl-2 antisense oligonucleotide therapy revealed an inverse correlation between chemosensitivity of melanoma cells and bcl-2 levels (Jansen *et al*, 1998). These findings indicate that the bcl-2 protein levels contribute to drug resistance. Our investigations of a bcl-2 overexpressing A375 cell line revealed that bcl-2 overexpression could not abolish the apoptosis triggered by pamidronate. This proposes that pamidronate-induced apoptosis is a process independent of mitochondrial activation.

Nitrogen containing bisphosphonates were shown to inhibit the farnesyl diphosphate synthase probably by mimicking the diphosphate moiety (van Beek *et al*, 1999). They are therefore inhibitors of the synthesis of higher isoprenoids like geranylgeranyl diphosphate. The prenylation of monomeric G-proteins such as members of the Ras superfamily like Rho proteins was shown to be reduced by bisphosphonate treatment. Geranylgeranylation of these proteins is required for their proper membrane association and hence activity. Rho family proteins are engaged in cytoskeletal reorganisation and enhanced expression of several isoforms was observed in metastatic tumour cells (Fritz *et al*, 1999). Moreover, ectopic overexpression of the Rho protein RhoC in A375 melanoma cells was sufficient to create a highly metastatic phenotype (Clark *et al*, 2000). Therefore, the inhibition of Rho proteins might provide a possibility to reduce metastasis through interference with this pathway. The involvement of the inhibitory effect of pamidronate on isoprenoid biosynthesis in induction of apoptosis was tested using farnesol and geranylgeraniol to circumvent the blockade of geraniol synthesis. Geranylgeraniol was more potent in abolishing pamidronate induced-apoptosis than farnesol. Supplying geranylgeraniol reduced apoptosis by about 75%, suggesting geranylgeranylated proteins such as Rho proteins to be the main target of the pamidronate-effect. The participation of the mevalonate pathway in bisphosphonate-induced apoptosis was also demonstrated in mouse macrophages (Luckman *et al*, 1998) and human myeloma cells (Shipman *et al*, 1998). An alternative mechanism of action has been described for the non-amino bisphosphonate clodronate (Frith *et al*, 1997). The incorporation of bisphosphonates into ATP generates non-hydrolyzable toxic analogues. This pathway may also in part account for the action of nitrogen-containing bisphosphonates. However, in our experiments, clodronate in the same concentration range as pamidronate failed to induce apoptosis in melanoma cell lines

Nitrogen-containing bisphosphonates with a similar bone protective potency to pamidronate at concentrations of several magnitudes less than required for pamidronate treatment have been described (Rogers *et al*, 2000). Different bisphosphonates are now introduced in clinical practice. For the use as chemotherapy, a major drawback of bisphosphonates is their high affinity to hydroxyapatite on the bone surface, which makes them preferentially delivered to sites of increased bone formation or resorption. Animal studies revealed that the skeletal uptake of alendronate reaches 90% of peak values within 1 h of intravenous or oral administration (Lin *et al*, 1991). This could limit their therapeutic applicability to the treatment of bone metastasis. Nevertheless, the issue of a survival benefit is still controversial (Wingen *et al*, 1986; Hall and Stoica, 1994; Diel *et al*, 1998). Therefore, new derivates with an increased bioavailability are required to extend the therapeutic spectrum of bisphosphonates.

Our findings show for the first time that pamidronate has a direct effect on melanoma cells, which is not blocked by overexpression of bcl-2. This data suggests that nitrogen-containing bisphosphonates could be a promising novel therapeutic class for the treatment and/or prevention of melanoma metastases. Although our results suggest that pamidronate has no synergistic effect with the classical chemotherapeutical agents such as DTIC, possible alternative combinations of chemotherapy and bisphosphonates should be further investigated. Further studies are also necessary to elucidate the underlying mechanism of their proapoptotic effect in more detail and to establish the potential antitumoural role of different bisphosphonate derivatives in human melanoma.

## References

[bib1] AparicioAGardnerATuYSavageABerensonJLichtensteinA1998 In vitro cytoreductive effects on multiple myeloma cells induced by bisphosphonates Leukemia 12 220229 9519785 10.1038/sj.leu.2400892

[bib2] AznarSLacalJC2001 Rho signals to cell growth and apoptosis Cancer Lett 165 110 11248412 10.1016/s0304-3835(01)00412-8

[bib3] BeanMABloomBRHerbermanRBOldLJOettgenHFKleinGTerryWD1975 Cell-mediated cytotoxicity for bladder carcinoma: evaluation of a workshop Cancer Res 35 29022913 1157057

[bib4] BergstromJDBostedorRGMasarachiaPJReszkaAARodanG2000 Alendronate is a specific, nanomolar inhibitor of farnesyl diphosphate synthase Arch Biochem Biophys 373 231241 10620343 10.1006/abbi.1999.1502

[bib5] BruggenJFoghJSorgC1981 Tumor production in the nude mouse, fibrinolytic activity and cross- reactivity with antimelanoma sera of various human tumor cell lines J Cancer Res Clin Oncol 102 141152 7200096 10.1007/BF00410665 PMC12252689

[bib6] CareyTETakahashiTResnickLAOettgenHFOldLJ1976 Cell surface antigens of human malignant melanoma: mixed hemadsorption assays for humoral immunity to cultured autologous melanoma cells Proc Natl Acad Sci USA 73 32783282 1067619 10.1073/pnas.73.9.3278 PMC431008

[bib7] ClarkEAGolubTRLanderESHynesRO2000 Genomic analysis of metastasis reveals an essential role for RhoC Nature 406 532535 10952316 10.1038/35020106

[bib8] DielIJSolomayerEFCostaSDGollanCGoernerRWallwienerDKaufmannMBastertG1998 Reduction in new metastases in breast cancer with adjuvant clodronate treatment N Engl J Med 339 357363 9691101 10.1056/NEJM199808063390601

[bib9] FrithJCMonkkonenJBlackburnGMRussellRGRogersMJ1997 Clodronate and liposome-encapsulated clodronate are metabolized to a toxic ATP analog, adenosine 5′-(beta, gamma-dichloromethylene) triphosphate, by mammalian cells in vitro J Bone Miner Res 12 13581367 9286751 10.1359/jbmr.1997.12.9.1358

[bib10] FritzGJustIKainaB1999 Rho GTPases are over-expressed in human tumors Int J Cancer 81 682687 10328216 10.1002/(sici)1097-0215(19990531)81:5<682::aid-ijc2>3.0.co;2-b

[bib11] GiardDJAaronsonSATodaroGJArnsteinPKerseyJHDosikHParksWP1973 In vitro cultivation of human tumors: establishment of cell lines derived from a series of solid tumors J Natl Cancer Inst 51 14171423 4357758 10.1093/jnci/51.5.1417

[bib12] HallDGStoicaG1994 Effect of the bisphosphonate risedronate on bone metastases in a rat mammary adenocarcinoma model system J Bone Miner Res 9 221230 8140935 10.1002/jbmr.5650090211

[bib13] HughesDEWrightKRUyHLSasakiAYonedaTRoodmanGDMundyGRBoyceBF1995 Bisphosphonates promote apoptosis in murine osteoclasts in vitro and in vivo J Bone Miner Res 10 14781487 8686503 10.1002/jbmr.5650101008

[bib14] JansenBSchlagbauer-WadlHBrownBDBryanRNvan ElsasAMullerMWolffKEichlerHGPehambergerH1998 bcl-2 antisense therapy chemosensitizes human melanoma in SCID mice Nat Med 4 232234 9461199 10.1038/nm0298-232

[bib15] LaemmliUK1970 Cleavage of structural proteins during the assembly of the head of bacteriophage T4 Nature 227 680685 5432063 10.1038/227680a0

[bib16] LeeMVFongEMSingerFRGuenetteRS2001 Bisphosphonate treatment inhibits the growth of prostate cancer cells Cancer Res 61 26022608 11289137

[bib17] LinJHDugganDEChenIWEllsworthRL1991 Physiological disposition of alendronate, a potent anti-osteolytic bisphosphonate, in laboratory animals Drug Metab Dispos 19 926932 1686238

[bib18] LuckmanSPHughesDECoxonFPGrahamRRussellGRogersMJ1998 Nitrogen-containing bisphosphonates inhibit the mevalonate pathway and prevent post-translational prenylation of GTP-binding proteins, including Ras J Bone Miner Res 13 581589 9556058 10.1359/jbmr.1998.13.4.581

[bib19] RaisovaMBektasMWiederTDanielPEberleJOrfanosCEGeilenCC2000 Resistance to CD95/Fas-induced and ceramide-mediated apoptosis of human melanoma cells is caused by a defective mitochondrial cytochrome c release FEBS Lett 473 2732 10802053 10.1016/s0014-5793(00)01491-5

[bib20] RaisovaMHossiniAMEberleJRiebelingCWiederTSturmIDanielPTOrfanosCEGeilenCC2001 The Bax/Bcl-2 ratio determines the susceptibility of human melanoma cells to CD95/Fas-mediated apoptosis J Invest Dermatol 117 333340 11511312 10.1046/j.0022-202x.2001.01409.x

[bib21] RogersMJChiltonKMCoxonFPLawryJSmithMOSuriSRussellRG1996 Bisphosphonates induce apoptosis in mouse macrophage-like cells in vitro by a nitric oxide-independent mechanism J Bone Miner Res 11 14821491 8889848 10.1002/jbmr.5650111015

[bib22] RogersMJGordonSBenfordHLCoxonFPLuckmanSPMonkkonenJFrithJC2000 Cellular and molecular mechanisms of action of bisphosphonates Cancer 88 29612978 10898340 10.1002/1097-0142(20000615)88:12+<2961::aid-cncr12>3.3.co;2-c

[bib23] SenaratneSGPirianovGMansiJLArnettTRColstonKW2000 Bisphosphonates induce apoptosis in human breast cancer cell lines Br J Cancer 82 14591468 10780527 10.1054/bjoc.1999.1131 PMC2363380

[bib24] SerroneLHerseyP1999 The chemoresistance of human malignant melanoma: an update Melanoma Res 9 5158 10338334 10.1097/00008390-199902000-00007

[bib25] ShipmanCMCroucherPIRussellRGHelfrichMHRogersMJ1998 The bisphosphonate incadronate (YM175) causes apoptosis of human myeloma cells in vitro by inhibiting the mevalonate pathway Cancer Res 58 52945297 9850051

[bib26] ShipmanCMRogersMJApperleyJFRussellRGCroucherPI1997 Bisphosphonates induce apoptosis in human myeloma cell lines: a novel anti-tumour activity Br J Haematol 98 665672 9332325 10.1046/j.1365-2141.1997.2713086.x

[bib27] SuwaHOhshioGImamuraTWatanabeGAriiSImamuraMNarumiyaSHiaiHFukumotoM1998 Overexpression of the rhoC gene correlates with progression of ductal adenocarcinoma of the pancreas Br J Cancer 77 147152 9459160 10.1038/bjc.1998.23 PMC2151257

[bib28] TwissIMPasORamp-KoopmanschapWDen HartighJVermeijP1999 The effects of nitrogen-containing bisphosphonates on human epithelial (Caco-2) cells, an in vitro model for intestinal epithelium J Bone Miner Res 14 784791 10320527 10.1359/jbmr.1999.14.5.784

[bib29] van BeekEPietermanECohenLLowikCPapapoulosS1999 Farnesyl pyrophosphate synthase is the molecular target of nitrogen- containing bisphosphonates Biochem Biophys Res Commun 264 108111 10527849 10.1006/bbrc.1999.1499

[bib30] WiederTOrfanosCEGeilenCC1998 Induction of ceramide-mediated apoptosis by the anticancer phospholipid analog, hexadecylphosphocholine J Biol Chem 273 1102511031 9556584 10.1074/jbc.273.18.11025

[bib31] WingenFEichmannTManegoldCKrempienB1986 Effects of new bisphosphonic acids on tumor-induced bone destruction in the rat J Cancer Res Clin Oncol 111 3541 3949849 10.1007/BF00402773 PMC12253181

